# Fractal analyses: statistical and methodological innovations and best practices

**DOI:** 10.3389/fphys.2013.00097

**Published:** 2013-05-08

**Authors:** John G. Holden, Michael A. Riley, Jianbo Gao, Kjerstin Torre

**Affiliations:** ^1^Complexity Group, Department of Psychology, CAP center for Cognition, Action, and Perception, University of CincinnatiCincinnati, OH, USA; ^2^Department of Psychology, CAP Center for Cognition, Action, and Perception, University of CincinnatiCincinnati, OH, USA; ^3^PMB Intelligence LLCWest Lafayette, IN, USA; ^4^Department of Mechanical and Materials Engineering, Wright State UniversityDayton, OH, USA; ^5^State Key Laboratory of Non-linear Mechanics, Institute of Mechanics, Chinese Academy of SciencesBeijing, China; ^6^Movement to Health, University MontpellierMontpellier, France

Fractal statistics now routinely appear in the scientific literature. Examples originate from many disciplines, including aquatic sciences, biology, computer science, ecology, economics, geology, mathematics, medicine, neuroscience, physics, physiology, and psychology. This eBook provides a broad range of resources to support the application of fractal methods and theory in physiology and related disciplines. It is comprised of a set of research topic articles that appeared in the *Frontiers in Physiology* specialty section: Fractal Physiology. Our eBook chapters are organized along a loose continuum defined by the characteristics of the empirical measurements a given statistical technique is intended to confront.

At one end of the continuum are techniques designed for application to stochastic systems. van Rooij et al. (2013) describe histograms, probability distributions, and scaling distributions in fractal terms. The next step on the continuum concerns self-affine random fractals and methods intended for outcome measures that entail scale-invariant 1/*f* patterns or related patterns of temporal fluctuation. Stadnitski (2012) overviews several statistical procedures available for the analysis of fractal time-series measurements. Riley et al. (2012) discuss an adaptive fractal analysis that broadens the potential range of bio-signals that can be understood from a fractal perspective. Likewise, Marmelat et al. (2012) illustrate a relative roughness scale, helpful in determining the applicability of a monofractal description to a given signal. Wijnants et al. (2013) examines how of signal sampling rate artifacts influence spectrally derived scaling exponents. Hasselman (2013) discusses relationships among a set of common fractal time-series analyses, and advocates reliance on theory-driven predictions as a route to understanding the systems that yield empirical patterns. Eke et al. (2012) bridge the monofractal and multifractal frameworks with a special emphasis on the appropriate and accurate characterization of measured signals. Ihlen (2012) supplies a detailed tutorial on multifractal detrended fluctuation analysis.

The deterministic end of the statistical continuum emphasizes techniques used to investigate systems that express differentiable trajectories. Webber (2012) illustrates recurrence analysis on time-series derived from several multi-dimensional dynamic systems. Gao et al. (2012) introduces a very general analysis that is suitable for use on both stochastic and continuous measurements. Finally, Richardson et al. (2012) describe techniques that assess relative dynamic synchrony among multiple coupled oscillatory time-series. Taken together, the chapters offer a gamut of analytic strategies alongside contemporary expertise on how to best conduct and interpret the outcomes of fractal analyses.
